# N95^®^ filtering facepiece respirator contamination with SARS-CoV-2 following reuse and extended use

**DOI:** 10.1017/ice.2025.92

**Published:** 2025-08

**Authors:** James S. Ford, Ralph C. Wang, Benjamin Stephenson, Nida F. Degesys, Jahan Fahimi, Edward M. Fisher, Delbert Harnish, Courtney M.C. Jones, Susan Peterson, Efrat Rosenthal, Richard Rothmann, Manish N. Shah, Vaishal Tolia, Anna Q. Yaffee, Katherine N. Yoon, Maria C. Raven, for the ReuseN95 Group

**Affiliations:** 1 Department of Emergency Medicine, University of California, San Diego, San Diego, CA, USA; 2 Department of Emergency Medicine, University of California, San Francisco, San Francisco, CA, USA; 3 Applied Research Associates, Engineering Science Division, Respiratory Protection Center of Excellence, Panama City, FL, USA; 4 Philip R. Lee Institute for Health Policy Studies, University of California, San Francisco, San Francisco, CA, USA; 5 Centers for Disease Control and Prevention, National Institute for Occupational Safety and Health, National Personal Protective Technology Laboratory, Pittsburgh, PA, USA; 6 Department of Emergency Medicine, University of Rochester School of Medicine and Dentistry, Rochester, NY, USA; 7 Department of Emergency Medicine, Johns Hopkins University School of Medicine, Baltimore, MD, USA; 8 BerbeeWalsh Department of Emergency Medicine, University of Wisconsin-Madison, Madison, WI, USA; 9 Department of Emergency Medicine, Emory University School of Medicine, Atlanta, GA, USA

## Abstract

**Objective:**

During the COVID-19 pandemic, the United States Centers for Disease Control and Prevention provided strategies, such as extended use and reuse, to preserve N95 filtering facepiece respirators (FFR). We aimed to assess the prevalence of N95 FFR contamination with SARS-CoV-2 among healthcare personnel (HCP) in the Emergency Department (ED).

**Design:**

Real-world, prospective, multicenter cohort study. N95 FFR contamination (primary outcome) was measured by real-time quantitative polymerase chain reaction. Multiple logistic regression was used to assess factors associated with contamination.

**Setting:**

Six academic medical centers.

**Participants:**

ED HCP who practiced N95 FFR reuse and extended use during the COVID-19 pandemic between April 2021 and July 2022.

**Primary exposure:**

Total number of COVID-19-positive patients treated.

**Results:**

Two-hundred forty-five N95 FFRs were tested. Forty-four N95 FFRs (18.0%, 95% CI 13.4, 23.3) were contaminated with SARS-CoV-2 RNA. The number of patients seen with COVID-19 was associated with N95 FFR contamination (adjusted odds ratio, 2.3 [95% CI 1.5, 3.6]). Wearing either surgical masks or face shields over FFRs was not associated with FFR contamination, and FFR contamination prevalence was high when using these adjuncts [face shields: 25% (16/64), surgical masks: 22% (23/107)].

**Conclusions:**

Exposure to patients with known COVID-19 was independently associated with N95 FFR contamination. Face shields and overlying surgical masks were not associated with N95 FFR contamination. N95 FFR reuse and extended use should be avoided due to the increased risk of contact exposure from contaminated FFRs.

## Introduction

N95 filtering facepiece respirators (FFRs) help protect frontline healthcare personnel (HCP) from pathogens such as SARS-CoV-2.^1^ In healthcare settings, N95 FFRs are intended to be single-use products and historically have been discarded after close contact with a patient, particularly in optimally-resourced settings.^1^ During the COVID-19 pandemic, the United States (US) Centers for Disease Control and Prevention (CDC) provided N95 FFR conservation contingency and crisis strategies for circumstances when supply was low, including “extended use” (sustained wearing for multiple patient encounters without removal) and “limited reuse” (wearing for multiple patient encounters, with doffing between encounters).^2–5^

Previous studies have evaluated proxies for safety following N95 FFR reuse, such as respirator fit following consecutive donnings and doffings.^2,6–8^ Previous in-vitro studies have demonstrated SARS-CoV-2 stability on N95 FFRs, providing a plausible mechanism for occupational contact exposure during FFR donning and doffing.^9,10^ One single-center, observational clinical study sampled surgical masks and face shields overlying N95 FFRs as a proxy for N95 FFR contamination, but this study did not directly test N95 FFR contamination.^11^ Many HCP practiced reuse and extended use of FFRs during the COVID-19 pandemic, but the safety of this practice has not been well-evaluated in a clinical setting.^12^ No real-world, clinical studies have been performed that directly examine the prevalence of N95 FFR contamination with SARS-CoV-2 following N95 FFR reuse and extended use.

In this study, we aimed to assess the prevalence of N95 FFR contamination with SARS-CoV-2 and identify factors associated with contamination following clinical use in the emergency department (ED).

## Methods

### Study design and setting

We conducted a prospective cohort study of HCP at six EDs in the United States.^8^ HCP were enrolled between April 2021 and July 2022 at the University of California-San Francisco, Emory University, Johns Hopkins University, the University of California-San Diego, the University of Rochester, and the University of Wisconsin-Madison. The study was reviewed and approved by the Western Copernicus Group Institutional Review Board (IRB) and the IRB at each participating site. All participants provided written informed consent. We adhered to STROBE reporting guidelines.^13^

### Study participants

Study participants were recruited via email correspondence at each study site. We enrolled HCP, including nurses, physicians, patient care technicians, pharmacists, and advanced practice providers (APPs: nurse practitioners, physician assistants) who practiced N95 FFR reuse or extended use as part of their clinical duties. We excluded individuals who repeatedly failed baseline fit testing, were unwilling to wear N95 FFRs for most of their shift, and had facial hair or jewelry that interfered with the face seal region of an N95 FFR.^14^ Pregnant clinicians were excluded from the study as they were strongly encouraged to not practice reuse or extended use.

### Study exposure and key variables

Variables collected were self-reported by HCP and included participant age, gender, race, ethnicity, clinical role (eg, physician, nurse), the total number of patients treated with confirmed SARS-CoV-2 (with the same FFR), hours the N95 FFR was worn, number of shifts the N95 FFR was worn, whether barrier adjuncts were worn over the N95 FFR (ie, surgical masks, face shields), and the total number of aerosol-generating procedures (AGPs) (eg, intubation, cardiopulmonary resuscitation) participated in by the HCP (with the same FFR). The primary exposure was the total number of COVID-19-positive patients treated by a healthcare worker. The primary outcome was N95 FFR contamination, defined by the presence of SARS-CoV-2 RNA, as detected by real-time quantitative polymerase chain reaction (RT-qPCR).

### Study procedures

Participants wore NIOSH-approved N95 FFRs that were available at their institution (Supplement A). The external components of these N95 FFRs were composed of polypropylene (3M: 1870/1870+, 1860, 1860 S, 92050), polyester (3M: 8210), or “SO SOFT Fabric” proprietary material (Halyard: 46727, 46867, 76827).^15–22^ Participants wore their N95 FFR until heavy soiling, deformation, fit failure, or five shifts had elapsed.^7^ This closely aligned with real-world use of N95 FFRs at the time, but differed from suggested CDC strategies (no more than 5 donnings, rather than 5 clinical shifts).^12,23^ However, FFR fit was assessed daily (after each shift), which is a practice suggested by the CDC when FFRs were donned more than 5 times.^23^ Between shifts, N95 FFRs were stored in individual plastic containers, then shipped to the Applied Research Associates (ARA) laboratory (Engineering Science Division, Panama City, Florida) to conduct SARS-CoV-2 testing using RT-qPCR (Supplement B). The time between FFR collection and shipping receipt at ARA was usually between 3 and 5 business days.

Upon receipt at ARA, N95 FFRs were stored at –80°C until sample testing could be performed. The mean time samples were stored before testing was 145 ± 74 days; the long-term stability of SARS-CoV-2 respiratory samples has been previously validated.^24^ The average time spent in storage and the variation between samples is larger than anticipated due to the need to optimize the virus extraction protocol after the start of testing, as well as shortages in reagents and consumables caused by the pandemic.

Coupon samples were taken from five locations on the N95 FFR. A 33-mm hole punch was used to sample each of the four quadrants of the N95 FFR body, and the straps were collected as one sample. FFRs were initially tested in the order in which they were received. However, we were unable to test all FFRs before the contract with ARA expired; thus, we randomly sampled and tested as many remaining FFRs as possible. To assess for sampling bias, we constructed histograms of FFR sampling for testing by “included” and “excluded” status and compared participant characteristics between included and excluded cohorts.

### Statistical analysis

We summarized data using descriptive statistics. We compared baseline characteristics between groups using Mann–Whitney *U* and Fischer’s exact tests. We employed a DAG-informed, multiple logistic regression model to assess factors associated with SARS-CoV-2 positivity (Supplement C & D). The primary exposure was the total number of COVID-19-positive patients a healthcare worker treated (continuous variable). We adjusted for number of AGP, age, ED role (physician vs non-physician), hours N95 FFR was worn, wearing a surgical mask over an FFR, and wearing a face shield over an FFR. We also performed a sensitivity analysis with an expanded model that included both material type and study site.

## Results

### Participant characteristics

We collected 412 N95 FFRs from 412 healthcare workers. Of these, 245 FFRs were tested for SARS-CoV-2 and were included in the final analysis (Supplement E). The mean age of participants in the final cohort was 36 ± 9 years and 62% (151/245) were women (Table 1). Physicians (45%, 111/245) comprised the largest group of participants, followed by nurses (24%, 58/245), advanced practice providers (APPs; 18%, 44/245), “other” (respiratory technicians and pharmacists; 7%, 18/245), and patient care technicians (PCTs; 6%, 14/245). HCP doffed their respirators 5.6 (± 0.3) times per shift on average. There were 453 AGP encounters (mean: 1.8/HCP). Eleven percent (27/245) of HCP accounted for over half (54%, 247/453) of all AGP encounters.

**Table 1.** Participant characteristics by COVID-19 exposure status

Characteristic	Total (n = 245)	Known COVID-19 exposure (n = 133)	No known COVID-19 exposure (n = 112)	*P*
Mean age (± SD)	36 (± 9)	36 (± 10)	36 (± 9)	1.0
Sex (n, %)^a^				
Female	151 (62%)	82 (62%)	69 (62%)	1.0
Race (n, %)				
Asian	27 (11%)	14 (11%)	13 (12%)	.97
Black or African American	38 (16%)	21 (16%)	17 (15%)	
White	160 65%	86 (65%)	74 (66%)	
2 or more races	11 (4%)	6 (4%)	5 (5%)	
Unknown	9 (4%)	6 (4%)	3 (3%)	
Ethnicity^b^ (n, %)				
Hispanic	18 (7%)	7 (5%)	11 (10%)	.22
HCP Type (n, %)				
Physician	111 (45%)	65 (49%)	46 (41%)	.56
Nurse	58 (24%)	28 (21%)	30 (27%)	
APP	44 (18%)	21 (16%)	23 (21%)	
Other	18 (7%)	10 (8%)	8 (7%)	
Patient Care Technician	14 (6%)	9 (7%)	5 (4%)	
N95 FFR Model (n, %)				
3M 1870	55 (22%)	23 (17%)	32 (29%)	.10
3M 1860	68 (28%)	44 (33%)	24 (21%)	
3M 1860 S	48 (20%)	32 (24%)	16 (14%)	
3M 8210	11 (4%)	5 (4%)	6 (5%)	
3M 9205	9 (4%)	4 (3%)	5 (5%)	
Halyard 46727	30 (12%)	15 (11%)	15 (13%)	
Halyard 46767	8 (4%)	3 (2%)	5 (5%)	
Halyard 46827	13 (5%)	5 (4%)	8 (7%)	
Halyard 76827	3 (1%)	2 (2%)	1 (1%)	
Total Doffings per Shift	5.7 (± 5.2)	5.8 (± 4.9)	5.5 (± 5.4)	.16
Wore Surgical Mask over N95 FFR	107 (44%)	71 (53%)	36 (32%)	.001
Wore Face Shield over N95 FFR	64 (26%)	41 (31%)	23 (21%)	.08
Wore Surgical Mask & Face Shield over N95 FFR	35 (14%)	23 (17%)	12 (11%)	.2
Total Hours Worn	23.4 (± 14)	27.5 (± 15.2)	18.5 (± 11.3)	<.001
Total Shifts Worn	2.3 (± 1.3)	2.7 (± 1.3)	1.9 (± 1.1)	<.001
Total AGPs	1.8 (± 3.1)	2.5 (± 3.6)	1.1 (± 2.4)	<.001
CPR Encounters	0.1 (± 0.5)	0.2 (± 0.6)	0.1 (± 0.4)	.17
Intubation Encounters	0.6 (± 1.4)	0.9 (± 1.7)	0.3 (± 0.8)	.006
Suctioning Encounters	0.4 (± 1.4)	0.5 (± 1.2)	0.3 (± 0.9)	.22
Nebulization Encounters	0.6 (± 1.4)	0.9 (± 1.7)	0.3 (± 0.7)	.008
NP Scope Encounters	0.1 (± 0.3)	0.1 (± 0.3)	0.1 (± 0.3)	.89

a Sex data missing for 1 patient.

b Ethnicity data missing for 2 patients.

AGP, aerosolizing generating procedures; APP, advanced practice provider (nurse practitioner, physician assistant); HCP, healthcare worker; n, number; SD, standard deviation. Comparisons between groups performed using Mann–Whitney and Fischer’s Exact tests, as appropriate.

Most participants (54%, 133/245) reported taking care of at least one COVID-19-positive patient during the study period. Participants with known exposure to COVID-19 were more likely to wear a surgical mask over their N95 FFR (53% vs 32%, *P* < .001), participated in more AGPs on average (2.5 vs 1.1, *P* < .001), and wore their FFRs for more total hours (28 vs 19 hours, *P* < .001) and more shifts (2.7 vs 1.9 shifts, *P* < .001), compared to those with no known exposure to COVID-19.

To assess for sampling bias, we compared participant characteristics by N95 FFR inclusion status. Excluded participants were statistically more likely to be physicians (56% vs 45%), and were less likely to wear a face shield over their N95 (11% vs 26%) or a face shield together with a surgical mask (3% vs 14%). Excluded participants also performed fewer nebulizing procedures on average (0.5 vs 0.6) compared to included participants (Supplement F). We constructed histograms comparing when a respirator was collected by N95 FFR inclusion/exclusion status and found that N95 FFRs were sampled reasonably evenly by time of collection, apart from the time period with the largest number of N95 FFRs collected (Supplement G).

### SARS-CoV-2 PCR testing results

**Figure 1.**
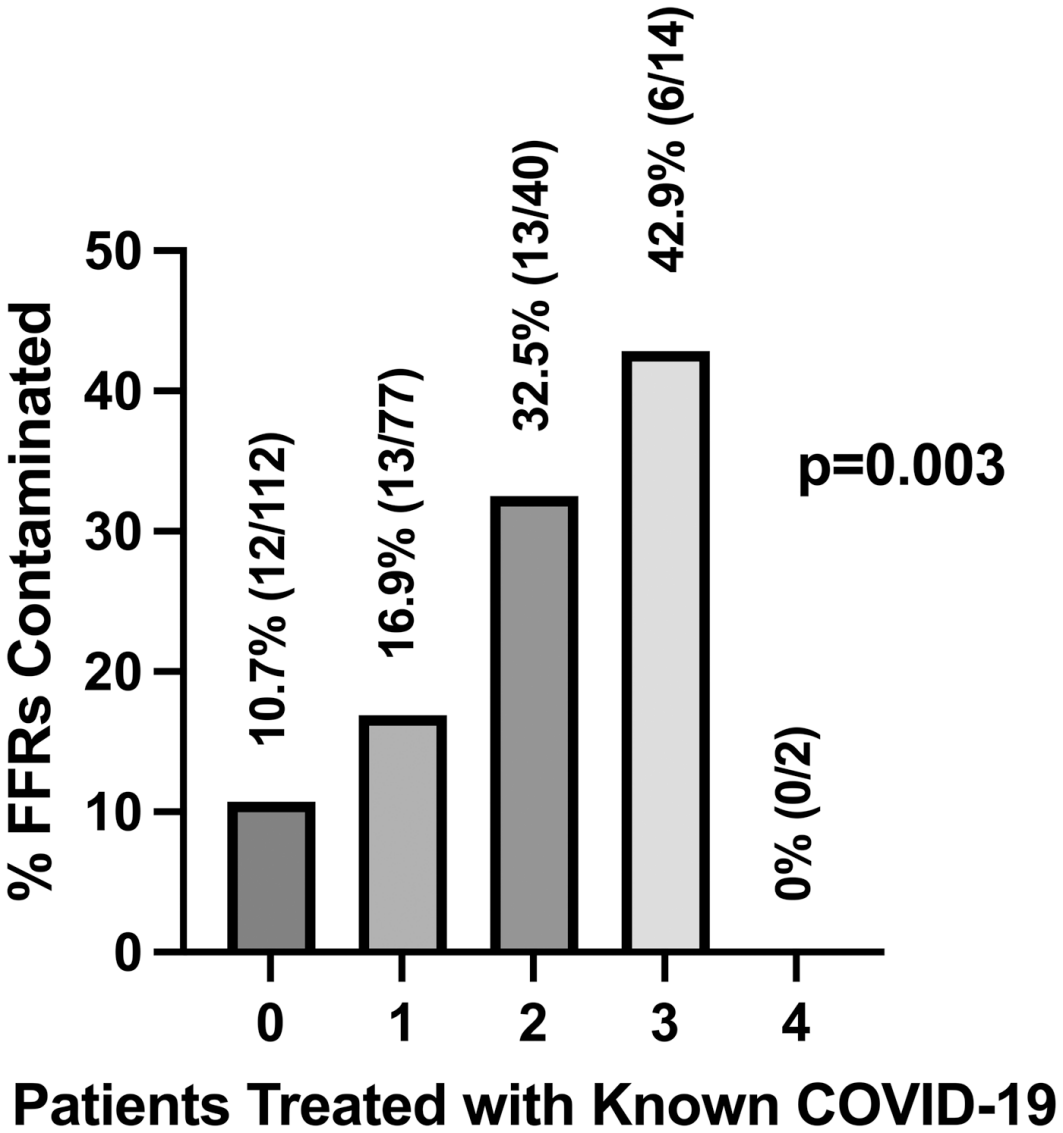
Prevalence of filtering facepiece respirator (FFR) contamination with SARS-CoV-2 by the number of known COVID-19-positive patients a healthcare worker treated while wearing that FFR.

Forty-four N95 FFRs (18.0%) tested positive for SARS-CoV-2 (mean PCR quantification cycles (cq) = 36.0, SD ± 2.2). Seventy-three percent (32/44) of contaminated N95 FFRs came from HCP who reported treating at least one patient with known COVID-19. FFR contamination increased as the number of known COVID-19 patients treated increased (Figure 1). FFR contamination was more common when HCP had known COVID-19 exposures (24%, 32/133) compared to when they did not (11%, 12/112). N95 FFR contamination was more common when a provider wore *either* a surgical mask *or* a face shield adjunct over the N95 FFR (either adjunct: 23%, no adjunct: 12%, *P* = .03) (Supplement H). The N95 FFR facepiece body was more often contaminated than the strap [body-only: 50% (22/44); strap-only: 32% (14/44); both body and strap: 11% (5/44)]. Contamination prevalence varied by institution, FFR model, and FFR material (Supplement A).

### Factors associated with N95 FFR SARS-CoV-2 contamination

In our adjusted model, total patients treated with confirmed COVID-19 was associated with FFR contamination (aOR, 2.3 [95% CI 1.5, 3.6], *P* < .001) (Table 2). Wearing a surgical mask or a face shield over the FFR was *not* associated with FFR contamination. Increasing age had a weak, negative association with FFR contamination (aOR 0.96 [95% CI 0.92, 1.0], *P* = .04).

**Table 2.** Unadjusted and adjusted logistic odds ratios for factors associated with N95 FFR contamination

Predictive factor	Unadjusted OR (95% CI)	Adjusted OR (95% CI)	*P*-value
Total Patients Treated with COVID-19	1.42 (0.96, 2.10)	2.32 (1.50, 3.58)	<.001
Age (10 years)^a^	0.56 (0.32, 1.00)	0.64 (0.92, 1.0)	.04
Healthcare Worker Type			
Physician (ref)	–		
Non-Physician	0.81 (0.36, 1.83)	1.10 (0.53, 2.28)	.8
Total Hours FFR Worn	1.00 (0.97, 1.03)	0.97 (0.94, 1.00)	.06
Total AGPs	0.96 (0.82. 1.11)	0.94 (0.82. 1.07)	.37
Surgical Mask over FFR	1.53 (0.79, 2.94)	1.36 (0.68, 2.74)	.39
Face Shield over FFR	1.82 (0.91, 3.65)	1.67 (0.78, 3.55)	.18

a Age analyzed as continuous variable with output reported as 10-year ORs.

AGP, aerosolizing generating procedure; aOR, adjusted odds ratio; HIV, human immunodeficiency virus; Ref, reference variable.

### Sensitivity analysis

To ensure that institutional differences in local COVID-19 prevalence, as well as the availability of FFR type, were not confounding our results, we created an expanded multiple logistic regression model that included site (blinded and by site with the lowest prevalence of FFR contamination as a reference group), as well as FFR material (material with the lowest prevalence of FFR contamination as a reference group). The total number of COVID-19-confirmed patients treated remained positively associated with FFR contamination (aOR 2.51 [95 % CI 1.58, 3.99], *P* < .001), whereas the inverse association with age was no longer significant (Supplement I). The FFR model was not associated with contamination. Several institutions were associated with contamination, but the CIs for the point estimates were wide.

## Discussion

In this multicenter cohort of frontline HCP, we provide the first real-world data on the prevalence of N95 FFR contamination with SARS-CoV-2 and risk factors for N95 FFR contamination. We found that caring for COVID-19-confirmed patients and increasing age were independently associated with contamination.

A systematic review reported nine distinct risk factor categories for contracting COVID-19 amongst HCP, including exposure to infected patients, lack of PPE, suboptimal hand hygiene, lack of infection control training, work overload, mental stress, cross-infection, unsafe disposal of medical waste, and inadequate disinfection.^25^ Among these nine, exposure to infected patients was considered the most important.^25^

In our study, HCP who reported treating a COVID-19-positive patient wore their N95 FFRs for more hours and more shifts on average than those who did not. However, when adjusting for total hours worn, we still saw a dose-dependent association between the number of patients a provider treated with COVID-19 and the odds of FFR contamination. This is likely explained by a higher number of SARS-CoV-2 particles in isolation rooms compared to the ambient ED environment. Nonetheless, N95 FFR contamination was high even among HCP with no known COVID-19 exposure (8%), which suggests that providers may have been unaware of patients’ COVID-19 positivity, may have been exposed by sources other than patients such as visitors or other HCP, or contamination may have been on the inside of the mask from asymptomatic COVID-19 infections in HCP.

In our study, increasing age of HCP had a weak negative association with N95 FFR contamination with SARS-CoV-2. This may relate to how EDs in teaching hospitals function. Often, resident physicians (who tend to be younger) are at the bedside more frequently and perform procedures that facilitate exposure. Conversely, older HCP may have spent less time at the bedside and often supervise, rather than perform procedures. This may have decreased exposure to SARS-CoV-2. Advanced age has been repeatedly associated with increased risk of severe COVID-19, hospitalization, and death,^26,27^ and frontline HCP’s fear of contracting COVID-19 has been shown to increase with increasing age.^28,29^ In our study, a lower proportion of N95 FFR contamination in older HCP may also have been driven by behavioral changes that minimized exposure. We found no association between participation in AGPs and N95 FFR contamination. However, there were only 1.8 AGP encounters per HCP on average, making it an infrequent risk event. Furthermore, over 50% of AGP encounters occurred in approximately 10% of HCP. This is consistent with ED staffing models, where certain providers who work in high-acuity ED zones are more likely to encounter patients ill enough to require an AGP. This may have aggregated risk in a small proportion of individuals, which may be why we see no association. We also saw no association between the number of hours N95 FFRs were worn and N95 FFR contamination. Nonetheless, overall N95 FFR contamination was common (11%), which likely increases the probability that HCP might be exposed to viral particles on the N95 FFR during repeated donning and doffing, which was also common in our study (∼6 times per shift).

One previous study that used contamination of overlying surgical masks and face shields as a proxy for N95 FFR contamination with SARS-CoV-2 found that 15% of surgical masks and face shields were contaminated, but this study did not directly measure N95 FFR contamination prevalence.^11^ In our study, surprisingly, wearing either surgical masks or face shields over FFRs was not independently associated with FFR contamination. This finding may be explained by HCP preferentially using surgical masks and face shields over FFRs in encounters with patients with known or suspected COVID-19, or in high risk encounters (eg AGPs). Additionally, our study showed that FFR straps were often contaminated. Practicing hand hygiene before and after respirator doffing may reduce contamination of FFR straps and contamination of HCP’s hands from contaminated straps, respectively.^9^

### Limitations

Many variables were self-reported, which gives the potential for participant recall bias. Due to unforeseen budgetary limitations, we were unable to test all planned N95 FFRs for SARS-CoV-2; thus, initial respirators were tested in the order they were received, and subsequent respirators were randomly selected for testing. We constructed histograms that plotted N95 FFR sampling frequency over time and found that the distribution of excluded respirators generally corresponded to the total number of respirators sampled per month. HCPs with included FFRs were more likely to wear surgical masks and face shields compared to HCP with excluded FFRs, were less likely to perform nebulizing procedures, and were more likely to be physicians, which likely represents a sampling bias. We do not know if the detected viral particles were on the inside, outside, or both sides of the FFR. Also, we do not know if the virus adhered to an FFR can be remobilized from the FFR, or if the virus is viable, making it difficult to assess the true risk of infection from FFR contact exposure. Study participants were not evenly distributed across sites, which may have introduced bias. While we performed a sensitivity analysis that included study site, unmeasured study site or participant characteristics may have influenced our results. A clinical outcome such as cases of COVID-19 among study participants would have been helpful for contextualizing the clinical impact of our findings.

Caring for patients with known COVID-19 was independently associated with N95 FFR contamination. Face shields and surgical masks did not help prevent respirator contamination; thus, we caution against reusing N95 FFRs when COVID-19 exposure has occurred, even when barrier adjuncts are employed. FFR straps can serve as a potential source of contact exposure; therefore, practicing hand hygiene before and after FFR doffing ostensibly reduces the potential for self-contamination.

## Supporting information

Ford et al. supplementary material Ford et al. supplementary material

## Data Availability

Data will be shared upon reasonable request of the study’s corresponding author, Dr. Ralph Wang.

## Supplementary material

The supplementary material for this article can be found at https://doi.org/10.1017/ice.2025.92

## Group information

Members of the Reuse N95 Group are listed in Supplement J.

## Meeting presentation

This study was presented in part at the American College of Emergency Physicians (ACEP) annual meeting; November 9, 2023; Philadelphia, PA.
