# Supplementary material for: N95® filtering facepiece respirator contamination with SARS-CoV-2 following reuse and extended use
Source: Infect Control Hosp Epidemiol. 2025 Jul 14;46(8):825–30. doi: 10.1017/ice.2025.92 (PMC12483621; doi:10.1017/ice.2025.92)
Supplement: Ford et al. supplementary material [file S0899823X25000923sup001.docx]

**Supplemental Materials**

**Table of Contents.**

| **Page** | **Title** |
| --- | --- |
| 2 | **A**. FFR contamination with SARS-CoV-2 by Site and FFR Model and Material |
| 3 | **B.** Full SARS-CoV-2 Testing Procedures for N95 FFRs |
| 5 | **C.** Directed Acyclic Graph |
| 6 | **D.** Justification of Multiple Logistic Regression Model |
| 7 | **E.** Participant inclusion and N95 FFR inclusion flow diagram |
| 8 | **F.** Comparison of participant characteristics between final cohort and excluded cohort |
| 10 | **G.** Sampling Distribution of FFRs According to Date Collected |
| 11 | **H**. FFR Contamination with SARS-CoV-2 by Sub-Groups |
| 13 | **I**. Sensitivity Analysis |
| 14 | **J.** Non-author collaborators from ReUseN95 Group |
| 15 | **K.** Supplement references |

**A. FFR contamination with SARS-CoV-2 by Study Site and FFR Model and Material**

Since the availability of FFR model varied by institution, we wanted to display FFR positivity prevalence by study site, FFR model, and FFR material **(Supplement Table 1).**

| **Supplement Table 1.** FFR contamination with SARS-CoV-2 by Site and N95 FFR Model and Material | | | | | | | |
| --- | --- | --- | --- | --- | --- | --- | --- |
|  | **Medical Center** | | | | | | |
| **FFR Material/Model** | **A** | **B** | **C** | **D** | **E** | **F** | **All** |
| **Polypropylene** | 9.7% (3/31) | 30% (15/50) | 44.4% (4/9) | 8.7% (2/23) | 18.6% (8/43) | 29.2% (7/24) | 21.7%  (39/180) |
| 3M 1870^10^ | - | 35.7% (5/14) | - | 6.7% (1/15) | 16.7% (2/12) | 21.4% (3/14) | 20% (11/55) |
| 3M 1860^11^ | 13.3% (2/15) | 38.9% (7/18) | 50% (3/6) | 0% (0/4) | 12.5% (3/24) | 100% (1/1) | 23.5% (16/68) |
| 3M 1860S^11^ | 6.3% (1/16) | 16.7% (3/18) | 33.3% (1/3) | 25% (1/4) | 42.9% (3/7) | - | 18.8% (9/48) |
| 3M 9205^12^ | - | - | - | - | - | 33.3% (3/9) | 33.3% (3/9) |
| **Polyester** | - | 0% (0/8) | - | 0% (0/3) | - | - | 0% (0/11) |
| 3M 8210^13^ | - | 0% (0/1) | - | 0% (0/3) | - | - | 0% (0/11) |
| **“SO SOFT Fabric”** | 0% (0/21) | 0% (0/2) | 22.2% (4/18) | - | 7.7% (1/13) | - | 9.3% (5/54) |
| Halyard 46727^14^ | 0% (0/14) | - | 28.6% (4/14) | - | - | - | 14.3% (4/28) |
| Halyard 46767^14^ | - | 0% (0/1) | - | - | 11.1% (1/9) | - | 10% (1/10) |
| Halyard 46827^15^ | 0% (0/4) | 0% (0/1) | 0% (0/4) | - | 0% (0/4) | - | 0% (0/13) |
| Halyard 76827^16^ | 0% (0/3) | - | - | - | - | - | 0%  (0/3) |
| **All FFRs** | 5.8% (3/52) | 25% (15/60) | 29.6% (8/27) | 7.7% (2/26) | 16.1% (9/56) | 29.2% (7/24) | 18% (44/245) |

**B. Full SARS-CoV-2 Testing Procedures for N95 FFRs**

After completion of clinical wear, N95 FFRs were shipped in batches to a private research enterprise (Applied Research Associates’ [ARA] Engineering Science Division, Panama City, Florida) to conduct SARS-CoV-2 testing. The time between FFR collection and shipping receipt at ARA was usually between 3 and 5 business days. Upon receipt at ARA, N95 FFRs were stored at -80℃ until sample testing could be performed. The mean time samples were stored before testing was 145 ± 74 days; the long-term stability of SARS-CoV-2 respiratory samples has been previously validated.^17^ The average time spent in storage and the variation between samples is larger than anticipated due to the need to optimize the virus extraction protocol after the start of receiving and shortages in reagents and consumables caused by the pandemic.

Coupon samples were taken from five locations on the N95 FFR (**Supplement** **Figure 1**). A 33-mm hole punch was used to sample each of the four quadrants of the N95 FFR body, and the straps were collected as one sample.


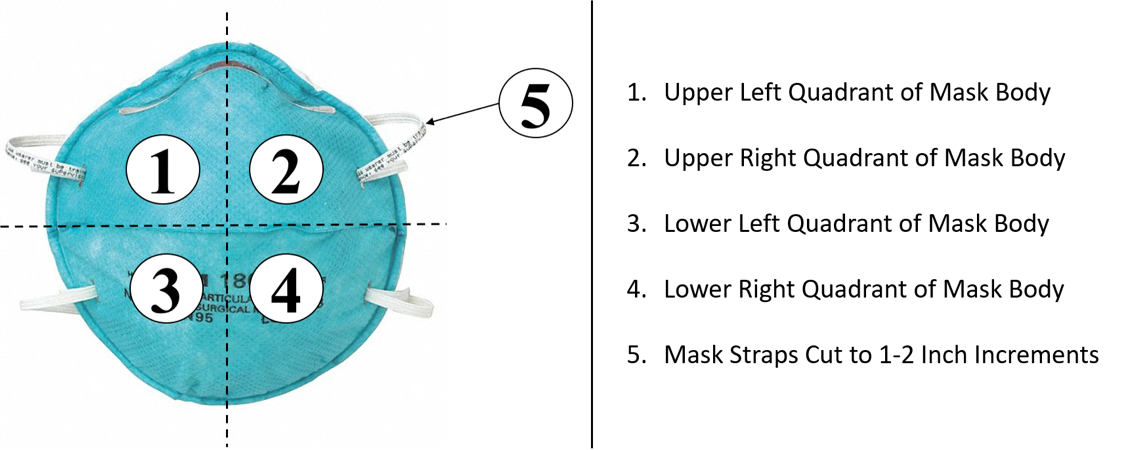


**Supplement Figure 1**. Locations of coupon sampling on N95 Filtered Facemask Respirators

**Sample coupon and strap collection locations**

Samples were placed into separate 50-mL centrifuge tubes (430921; Corning Inc.; Corning, NY) with 25 mL of extraction buffer (0.1% Tween 80 in 1X PBS) and 1 x 10^5^ copies of MS2 phage (10165948001; Roche Diagnostics; Mannheim Germany) to serve as an exogenous control. Virus was extracted by vortexing samples for 15 minutes at room temperature. The extraction method was validated for and compared against all FFR models used in this study and found to be repeatable and comparable. Samples were concentrated using 30MWCO PES protein concentrator tubes (88531; Pierce) and RNA extraction was performed using the Viral MagBead Kit (Zymo Research, Irvine, CA, USA).

SARS-CoV-2 RNA was detected via real-time quantitative polymerase chain reaction (RTqPCR) using the Research Use Only 2019-nCoV qPCR Probe Assay Kit (10006713; IDT; Coralville, IA), which utilizes two primer-Probe sets (2019-nCOV_N1 and 2019-nCOV_N2) to target separate regions of the SARS-CoV-2 Nucleocapsid gene (N). A primer-probe set that targeted the human RNase-P gene (RP) was used as an endogenous control for nucleic acid extraction. MS2 phage was used an exogenous control in samples that were spiked with 1 x 10^5^ copies of MS2 phage. MS2 RNA was detected using a primer-probe set adapted from a previous study targeting the RNA replicase gene. Primer-probe sets used for this study are listed below in **Supplement** **Table 2.**

| **Table 2. Primer-Probe Sets for SARS-CoV-2 Testing** | |
| --- | --- |
| **Primer-Probe Set Type** | **Primer-Probe Set Description** |
| N1 Primer-Probe Set | - - 2019-nCoV_N1-F: 5’ -GAC CCC AAA ATC AGC GAA AT- 3’   - 2019-nCoV_N1-R: 5’-TCT GGT TAC TGC CAG TTG AAT CTG- 3’   - 2019-nCoV_N1-P: 5’ -FAM-ACC CCG CAT TAC GTT TGG TGG ACC-BHQ1- 3’ |
| N2 Primer-Probe Set | - - 2019-nCoV_N2-F: 5’ -TTA CAA ACA TTG GCC GCA AA- 3’   - 2019-nCoV_N2-R: 5’-GCG CGA CAT TCC GAA GAA- 3’   - 2019-nCoV_N2-P: 5’ -FAM-ACA ATT TGC CCC CAG CGC TTC AG-BHQ1- 3’ |
| RP Primer-Probe Set | - - RP-F: 5’ -AGATTTGGACCTGCGAGCG – 3’   - RP-R: 5’ -GAGCGGCTGTCTCCACAAGT – 3’   - RP-P: 5’ -FAM- TTCTGACCTGAAGGCTCTGCGCG -BHQ1 – 3’ |
| MS2 Primer-Probe Set | - - MS2-F: 5’ -GCT CTG AGA GCG GCT CTA TTG – 3’   - MS2-R: 5’ -CGT TAT AGC GGA CCG CGT - 3’   - MS2-P: 5’ -Cy5- CC GAG ACC AAT GTG CGC CGT G-IAbRQsp – 3’ |

One-step RTqPCR was performed using the AriaMx Real-Time PCR System (Agilent Technologies, Santa Clara, CA) and the Brilliant II QRT-PCR master mix (600809; Agilent Technologies; Santa Clara, CA) using ROX as a passive reference dye. Reactions were performed in duplicate on 96-well plates using the thermal profile listed in **Supplement Table 3**.

| **Supplement Table 3. Thermal Profile used for RT-qPCR** | |
| --- | --- |
| **Step #** | **Step Description** |
| 1 | 30 min at 50 ℃ |
| 2 | 10 min at 95 ℃ |
| 3 | 15 sec at 95 ℃ |
| 4 | 1 min at 55 ℃ |
| 5 | Repeat Steps 3 – 4 for 40 cycles |

Each plate was run with a no template control (NTC) and a positive control containing 10^3^ copies of synthetic SARS-CoV-2 RNA (102024; Twist Bioscience; San Francisco, CA) or purified MS2 RNA. Each sample was tested for SARS-CoV-2 using the N1 and N2 primer-probe sets along with an endogenous or exogenous control (RP or MS2). Samples were classified as “positive” if at least one of the two targets (N1 or N2) produced a CT value of < 37. Samples were considered negative for SARS-CoV-2 if neither target was amplified and the endogenous (RP) or exogenous (MS2) control was detected. If RP or MS2 is not detected in a SARS-CoV-2 negative sample, the results were considered a false negative.

**C. Directed Acyclic Graph**

We created a directed acylic graph (DAG) meant to isolate the effect of the primary exposure “Total patients seen with COVID-19” on the primary outcome “N95 Contamination with SARS-CoV-2” (**Supplement Figure 2**).

**
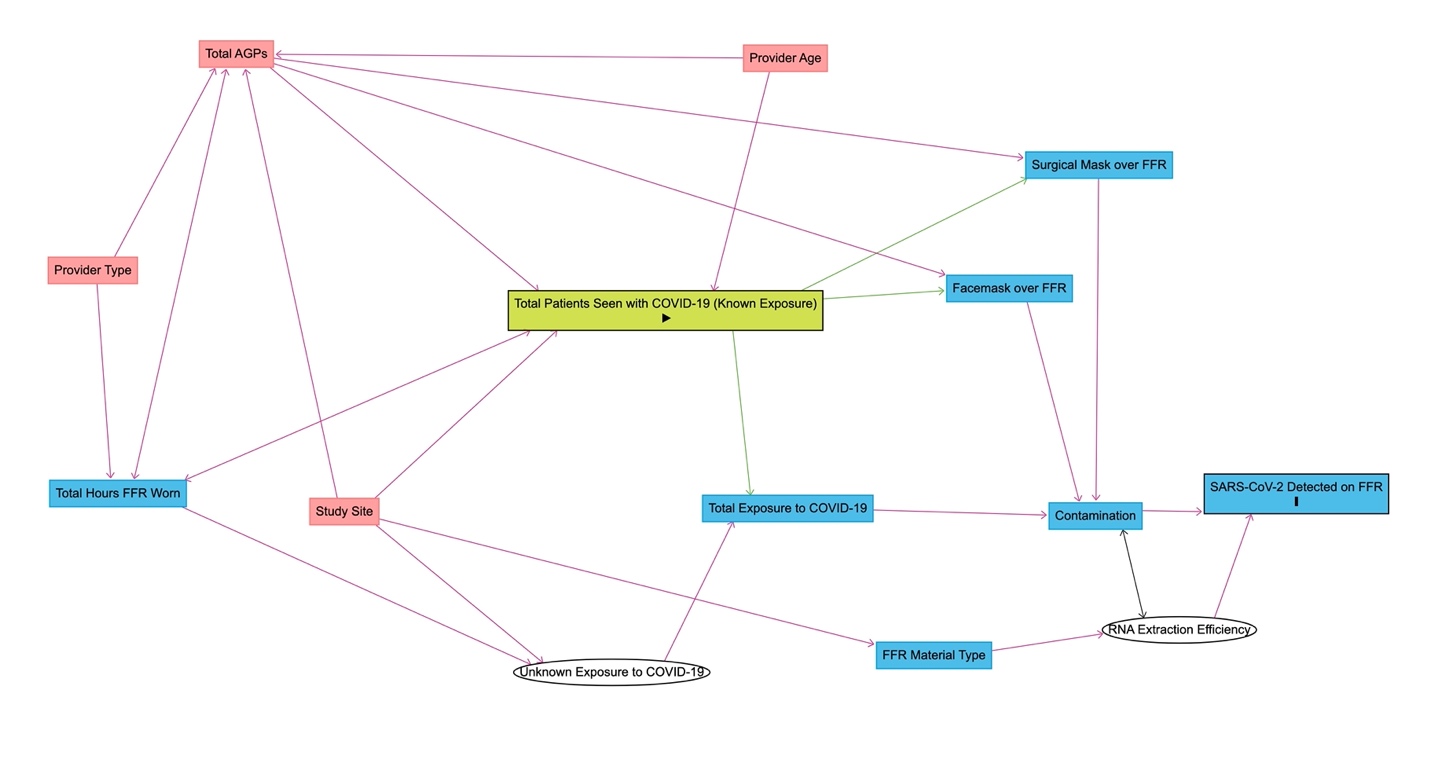
**

**Supplement Figure 2.** Directed acyclic graph. The primary outcome is “N95 Contamination with SARS-CoV-2”. The green box is the primary exposure of interest, “Total patients seen with COVID-19”. Blue boxes represent ancestors of the outcome. Pink boxes represent ancestors of both the exposure and the outcome. White boxes represent unobserved variables. DAG created on www.daggity.net.

**D. Justification of Multiple Logistic Regression Model**

We employed a multiple logistic regression model to assess factors associated with N95 FFR SARS-CoV-2 positivity. We constructed a DAG to conceptually map the complex relationships between variables and to help us choose which variables to include in the model. The primary exposure was total number of COVID-19 positive patients treated (continuous). Other factors that were felt to most plausibly be associated with SARS-CoV-2 positivity were included in the model, including number of aerosolizing procedures (AGP) performed by HCP, age of HCP, ED role (Physician vs. Other), total hours N95 FFR worn, wearing a surgical mask over an FFR, and wearing a face shield over an FFR. Age was included as a predictor because we hypothesized that older providers (e.g. attending physicians, charge nurse) would spend less time at the patients’ bedside. We report the regression output for age as a 10-year aOR, because clinical roles tend to change more substantively with each decade of clinical service. We included ED role as a predictor variable because these roles are likely to be associated with time spent in the patient’s room and proximity to the patient. As there were few outcomes, we made ED role a binary variable (physician, non-physician) so as not to overfit our model. We included total hours worn and not total shifts worn because the former is a more precise measure of potential time under exposure to SARS-CoV-2 and shift length was variable (e.g. 8 vs. 12 hours). We intentionally left the coefficient as the increase in odds per single hour because we felt that a single hour was a clinically meaningful time frame for exposure. As certain AGPs were more common than others, we aggregated AGPs into a single variable and used this as a predictor variable (continuous). We also intentionally left the “total AGPs” coefficient as increase in risk per single AGP encounter, because we felt that even a single AGP encounter was clinically meaningful. We included whether HCP used barrier adjuncts (surgical mask, face shield) over their FFRs as separate variables, as these were thought to provide protection against FFR contamination. As local disease prevalence likely varied by study site, we chose not to include study site in the primary analysis because of concerns with collinearity “total patients treated with COVID-19.” Additionally, availability of different FFR models (and therefore FFR material type) varied by study site, so we did not include FFR material type in our primary analysis due to concerns for collinearity, and because some FFR models were used very infrequently. However, we performed a sensitivity analysis with an expanded model that included both material type and study site.

**E. Participant inclusion and N95 FFR inclusion flow diagram**

**
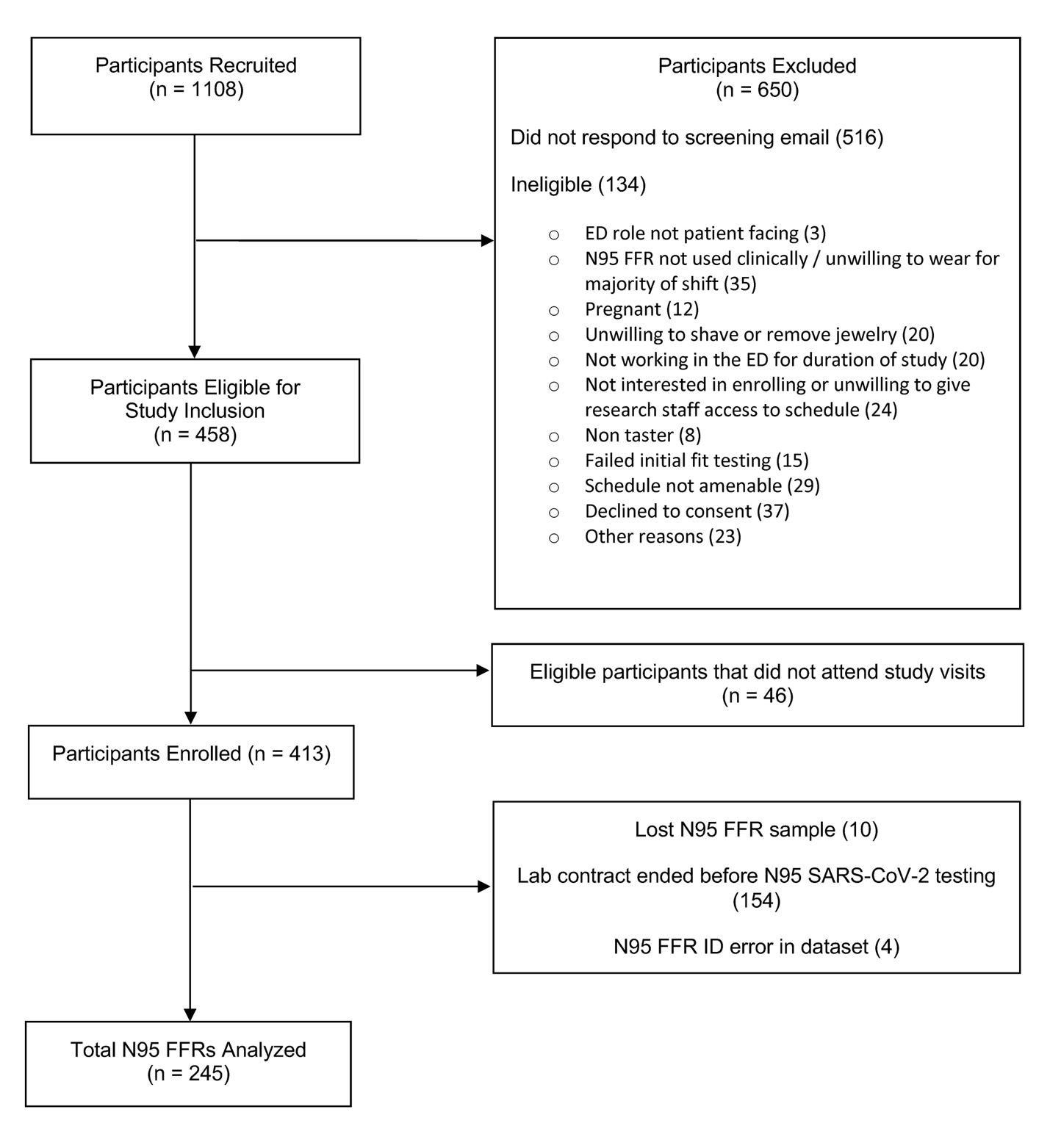
**

**Supplement Figure 3.** Flow diagram demonstrating inclusion of participants and their corresponding N95 filtering facemask respirator

**F. Comparison of Participant Characteristics Between Final Cohort and Excluded Cohort**

Note: To assess for sampling bias, we compared participant characteristics by N95 FFR inclusion status (**Supplement Table 4**). Excluded participants were statistically more likely to be physicians, were less likely to wear a surgical mask or face shield, and performed fewer nebulizing procedures compared to included participants.

| **Supplement Table 4.** Comparison of Participant Characteristics Between Final Cohort and Excluded Cohort | | | | |
| --- | --- | --- | --- | --- |
| **Characteristic** | **Total**  **(n=413)** | **Included**  **(n=245)** | **Excluded**  **(n=168)** | **P** |
| **Mean age** (± SD) | 37 (± 9) | 36 (± 9) | 37 (± 10) | 0.65 |
| **Sex** (n, %)**^1^** |  |  |  |  |
| Male | 39% (158/410) | 38% (93/244) | 39% (65/166) | 0.84 |
| Female | 61% (252/410) | 61.6% (151/244) | 61% (101/166) |  |
| **Race** (n, %) |  |  |  |  |
| Asian | 13% (54/408) | 11% (27/245) | 16% (27/168) | 0.061 |
| Black/African American | 13% (51/408) | 16% (38/245) | 7% (12/168) |  |
| White | 67% (275/408) | 65% (160/245) | 68% (115/168) |  |
| 2 or more races | 4% (16/408) | 4% (11/245) | 4% (6/168) |  |
| Unknown | 4% (17/413) | 4% (9/245) | 5% (8/168) |  |
| **Ethnicity^2^** (n, %) |  |  |  |  |
| Hispanic | 8% (33/408) | 7% (18/243) | 9% (15/165) | 0.58 |
| Not Hispanic | 92% (375/408) | 93% (225/243) | 91% (150/165) |  |
| **HCP Type^3^** (n, %) |  |  |  |  |
| Physician | 50% (285/412) | 45% (111/245) | 56% (94/167) | 0.001 |
| Nurse | 25% (103/412) | 24% (58/245) | 27% (45/167) |  |
| APP | 13% (52/412) | 18% (44/245) | 5% (8/167) |  |
| Other^4^ | 7% (30/412) | 7% (18/245) | 7% (12/167) |  |
| Patient Care Technician | 5% (22/412) | 6% (14/245) | 5% (8/167) |  |
| **N95 FFR Model^5^** (n, %) |  |  |  |  |
| 3M 1870 | 29% (121/412) | 22% (55/245) | 40% (66/167) | 0.94 |
| 3M 1860 | 22% (89/412) | 28% (68/245) | 13% (21/167) |  |
| 3M 1860 S | 16% (66/412) | 20% (48/245) | 11% (18/167) |  |
| 3M 8210 | 4% (16/412) | 4% (11/245) | 3% (5/167) |  |
| 3M 9205 | 5% (22/412) | 4% (9/245) | 8% (13/167) |  |
| Halyard 46727 | 13% (55/412) | 12% (28/245) | 15% (25/167) |  |
| Halyard 46767 | 4% (15/412) | 4% (10/245) | 4% (7/167) |  |
| Halyard 46827 | 6% (25/412) | 5% (13/245) | 7% (12/167) |  |
| Halyard 76827 | 1% (3/412) | 1% (3/245) | 0% (0/167) |  |
| **Total Doffings per Shift** | 6.1 (± 5.8) | 5.7 (± 5.2) | 6.7 (± 6.6) | 0.32 |
| **Wore Surgical Mask over N95 FFR** | 40% (165/413) | 44% (107/245) | 35% (58/168) | 0.07 |
| **Wore Face Shield over N95 FFR** | 20% (83/413) | 26% (64/245) | 11% (19/168) | **<0.001** |
| **Wore Surgical Mask or Face Shield over N95 FFR** | 51% (209/413) | 56% (136/245) | 43% (73/168) | **0.02** |
| **Wore Surgical Mask & Face Shield over N95 FFR** | 9% (39/413) | 14% (35/245) | 3% (4/168) | **<0.001** |
| **Total Hours Worn** | 22.7 (± 14.5) | 23.4 (± 14) | 21.6 (± 14.8) | 0.08 |
| **Total Shifts Worn** | 2.3 (± 1.3) | 2.3 (± 1.3) | 2.2 (± 1.2) | 0.21 |
| **Treated ≥1 patient(s) with COVID-19** | 53% (219/413) | 54% (133/245) | 51% (86/168) | 0.55 |
| **Total Patients Treated with COVID-19** | 0.8 (± 0.9) | 0.8 (± 0.9) | 0.7 (± 0.9) | 0.31 |
| **Total AGPs** | 1.9 (± 3.9) | 1.8 (± 3.1) | 1.9 (± 4.8) | 0.38 |
| CPR Encounters | 0.1 (± 0.5) | 0.1 (± 0.5) | 0.1 (± 0.4) | 0.82 |
| Intubation Encounters | 0.7 (± 1.6) | 0.6 (± 1.4) | 0.8 (± 1.8) | 0.56 |
| Suctioning Encounters | 0.4 (± 1.2) | 0.4 (± 1.4) | 0.4 (± 1.1) | 0.53 |
| Nebulization Encounters | 0.6 (± 1.5) | 0.6 (± 1.4) | 0.5 (± 1.6) | **0.04** |
| NP Scope Encounters | 0.1 (± 0.4) | 0.1 (± 0.3) | 0.1 (± 0.5) | 0.53 |
| AGP, aerosolizing generating procedures; APP, advanced practice provider (nurse practitioner, physician assistant); HCW, healthcare worker; n, number; SD, standard deviation. ^1^Sex data missing for 3 participants. ^2^Ethnicity data missing for 5 participants. ^3^HCW type data missing for 1 participant. ^4^Other group composed of respiratory technicians and pharmacists.  ^5^FFR model data missing for 1 participant. Comparisons between groups performed using Mann-Whitney U and Fischer’s Exact tests, as appropriate. | | | | |

**G. Sampling Distribution of FFRs According to Date Collected**

**Note**: Histograms comparing when a respirator was collected by N95 FFR inclusion/exclusion status showed that N95 FFRs were sampled reasonably evenly by time of collection, except for the time period with the largest number of N95 FFRs collected (**Supplement Figure 3**).

**
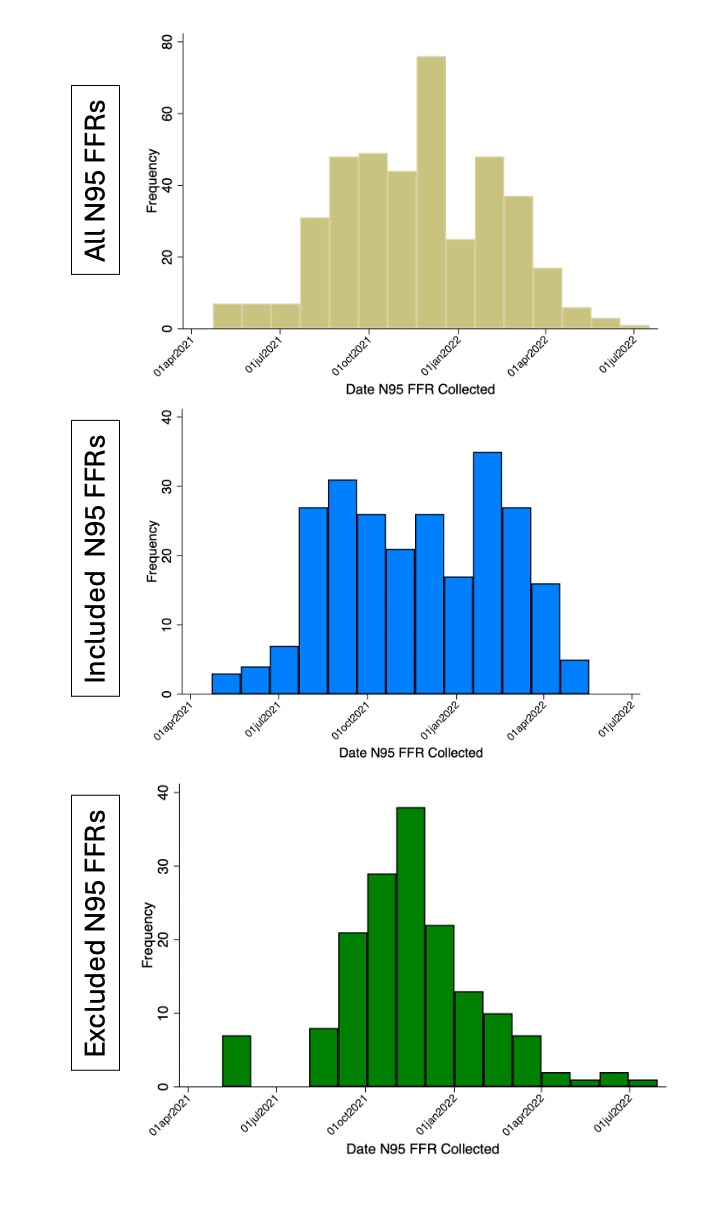
**

**Supplement Figure 4.** Histograms comparing when an N95 filtering facemask respirator was collected by inclusion/exclusion status

**H. N95 FFR Contamination with SARS-CoV-2 by Sub-Groups**

We provide proportions of contamination with SARS-CoV-2 according to participant and N95 filtering facemask respirator characteristics (**Supplement Table 5**).

| **Supplement Table 5.** Proportion of N95 filtering facemask respirator contamination according to sub-groups | | |
| --- | --- | --- |
| **Characteristic** | **% Contaminated (n=245)** | ***P*** |
| **Gender** |  |  |
| Male | 16.1% (15/93) | 0.7 |
| Female | 19.2% (29/151) |  |
| Refused/Unknown | 0% (0/1) |  |
| **Race** |  |  |
| Asian | 14.8% (4/27) | 0.8 |
| Black or African American | 13.2% (5/38) |  |
| White | 19.4% (31/160) |  |
| 2 or more races | 27.3% (3/11) |  |
| Unknown | 11.1% (1/9) |  |
| **Ethnicity** |  |  |
| Hispanic | 11.1% (2/11) | 0.3 |
| Not Hispanic | 18.7% (42/225) |  |
| **Health care worker type** |  |  |
| Physician | 17.1% (19/111) | 0.5 |
| Nurse | 22.4% (13/58) |  |
| Nurse Practitioner/Physician Assistant | 27.8% (5/18) |  |
| Other | 13.6% (6/44) |  |
| Patient Care Technician | 7% (1/14) |  |
| **N95 FFR Model** |  |  |
| 3M 1870 | 20% (11/55) | 0.3 |
| 3M 1860 | 24% (16/68) |  |
| 3M 1860 S | 19% (9/48) |  |
| 3M 8210 | 0% (0/11) |  |
| 3M 9205 | 33% (3/9) |  |
| Halyard 46727 | 14% (4/29) |  |
| Halyard 46867 | 10% (1/10) |  |
| Halyard 46827 | 0% (0/13) |  |
| Halyard 76827 | 0% (0/3) |  |
| **N95 Material** |  |  |
| Polypropylene (3M 1870/1860/1860S/9205) | 22% (39/180) | 0.03 |
| Polyester (3M 8210) | 0% (0/11) |  |
| SO SOFT Fabric (Halyard 46727, 46867, 46827, 76827) | 9% (5/54) |  |
| **Use of Surgical Mask Over N95 FFR** |  |  |
| Yes | 22% (23/107) | 0.2 |
| No | 15% (21/138) |  |
| **Use of Face Shield over N95 FFR** |  |  |
| Yes | 25% (16/64) | 0.1 |
| No | 15% (28/181) |  |
| **Wore Surgical Mask or Face Shield over N95 FFR** |  |  |
| Yes | 23% (31/136) | 0.03 |
| No | 12% (13/109) |  |
| **Wore Surgical Mask and Face Shield over N95 FFR** |  |  |
| Yes | 25% (16/64) | 0.5 |
| No | 15% (28/181) |  |
| **Study Site (Blinded)** |  |  |
| Site A | 6% (3/52) | 0.01 |
| Site B | 25% (15/60) |  |
| Site C | 30% (8/27) |  |
| Site D | 8% (2/26) |  |
| Site E | 16% (9/56) |  |
| Site F | 29% (7/24) |  |

| **I. Sensitivity Analysis**  We performed a sensitivity analysis including N95 filtering facemask respirator material and study site (**Supplement Table 6**).  **Supplement Table 6.** Sensitivity Analysis with Expanded Model with Unadjusted and Adjusted Logistic Odds Ratios for Factors Associated with N95 FFR Contamination | | | |
| --- | --- | --- | --- |
| **Predictive Factor** | **Unadjusted OR (95% CI)** | **Adjusted OR**  **(95% CI)** | **p-value** |
| **Total Patients Treated with COVID-19** | 1.42 (0.96, 2.10) | 2.51(1.58, 3.99) | <0.001 |
| **Age (10 years)^1^** | 0.56 (0.32, 1.00) | 0.68 (0.42, 1.12) | 0.13 |
| **Healthcare Worker Type** |  |  |  |
| Physician (ref) | - | - | - |
| Non-Physician | 0.81 (0.36, 1.83) | 1.02 (0.45, 2.28) | 0.97 |
| **Total Hours N95 FFR Worn** | 1.00 (0.97, 1.03) | 0.97 (0.94, 1.00) | 0.08 |
| **Total AGPs** | 0.96 (0.82. 1.11) | 0.94 (0.81. 1.08) | 0.39 |
| **Surgical Mask over N95 FFR^4^** | 1.53 (0.79, 2.94) | 1.57 (0.71, 3.45) | 0.71 |
| **Face Shield over N95 FFR^4^** | 1.82 (0.91, 3.65) | 1.48 (0.56, 3.86) | 0.43 |
| **FFR Material** |  |  |  |
| Polyester^2^ (ref)fa | - | - | - |
| Polypropylene^3^ | 2.7 (1.01, 7.27) |  | - |
| “SO SOFT” Fabric^4^ | * | * | * |
| **Study Site** |  |  |  |
| A (ref) | - | - | - |
| B | 5.44 (1.48, 20.06) | 4.32 (0.87, 21.32) | 0.073 |
| C | 6.88 (1.65, 28.70) | 18.93 (3.59, 99.9) | 0.001 |
| D | 1.36 (0.21, 8.70) | 1.64 (0.22, 12.42) | 0.634 |
| E | 3.13 (0.80, 12.27) | 4.71 (1.02, 21.67) | 0.047 |
| F | 6.73 (1.56, 28.98) | 5.96 (1.13, 31.36) | 0.035 |
| \| ^1^Age analyzed as continuous variable with output reported as 10-year ORs.^2^Polyester FFR (3M 8210). ^3^Polypropylene N95 FFR (3M 1870/1860/1860S/9205). ^4^SO SOFT Fabric N95 FFRs (Halyard 46727/46767/46827/76827). ^4^We also tested a model that replaced surgical mask and face shield variables with a combined “Surgical mask *and* face shield over N95 FFR” variable: aOR 1.4 (0.6, 3.6), results were otherwise similar. *Omitted due to issues with collinearity. AGP, aerosolizing generating procedure. aOR, adjusted odds ratio. HIV, human immunodeficiency virus. Ref, reference variable. \|  \| \| --- \| --- \| | | | |

**J. Non-author collaborators from ReUseN95 Group**

We include our non-author collaborators from the ReUseN95 Group in **Supplement Table 7.**

| **Supplement Table 7. Non-author collaborators from the ReUseN95 Group** | |
| --- | --- |
| **Full Name** | **Institution** |
| Anna R. Harris (MA) | Department of Emergency Medicine, University of California, San Francisco |
| Robin Kemball (MPH) | Department of Emergency Medicine, University of California, San Francisco |
| Alexis Mitchner (BA) | Department of Emergency Medicine, University of California, San Francisco |
| Angela Wong (BA) | Department of Emergency Medicine, University of California, San Francisco |
| Alex Hall (DHSc, MS, RN) | Department of Emergency Medicine, Emory University, Atlanta |
| Rabbiya Iqbal (BS) | Department of Emergency Medicine, Johns Hopkins University, Baltimore |
| Michael Kramer (BS) | Department of Emergency Medicine, Johns Hopkins University, Baltimore |
| Kendall Maliszewski (BS) | Department of Emergency Medicine, Johns Hopkins University, Baltimore |
| Breana McBryde (BS) | Department of Emergency Medicine, Johns Hopkins University, Baltimore |
| John DeAngelis (MD) | Department of Emergency Medicine, University of Rochester Medical Center |
| Emily Corbett-Valade (MS) | Department of Emergency Medicine, University of Rochester Medical Center |
| Edward Castillo (PhD, MPH) | Department of Emergency Medicine, University of California, San Diego |
| Apoorva Maru (BS, BA) | BerbeeWalsh Department of Emergency Medicine, University of Wisconsin, Madison |
| Angela Gifford (MA) | BerbeeWalsh Department of Emergency Medicine, University of Wisconsin, Madison |

**K. Supplement References**

1. NIOSH. NIOSH Guide to the Selection and Use of Particulate Respirators. 1996; <https://www.cdc.gov/niosh/docs/96-101/default.html>. Accessed 04/09/2023.

2. Fisher EM, Shaffer RE. Considerations for recommending extended use and limited reuse of filtering facepiece respirators in health care settings. *J Occup Environ Hyg.* 2014;11(8):D115-128.

3. CDC. Centers for Disease Control and Prevention: Summary for Healthcare Facilities: Strategies for Optimizing the Supply of N95 Respirators during Shortages. <https://www.jointcommission.org/-/media/tjc/documents/covid19/summary-strategies-to-optimize-the-supply-of-ppe-during-shortages-_-cdc.pdf>. Accessed 04/09/2023.

4. Emanuel EJ, Persad G, Upshur R, et al. Fair Allocation of Scarce Medical Resources in the Time of Covid-19. *N Engl J Med.* 2020;382(21):2049-2055.

5. Ranney ML, Griffeth V, Jha AK. Critical Supply Shortages - The Need for Ventilators and Personal Protective Equipment during the Covid-19 Pandemic. *N Engl J Med.* 2020;382(18):e41.

6. Kea B, Johnson A, Lin A, et al. An international survey of healthcare workers use of personal protective equipment during the early stages of the COVID-19 pandemic. *J Am Coll Emerg Physicians Open.* 2021;2(2):e12392.

7. Wang RC, Degesys NF, Fahimi J, et al. Incidence of Fit Test Failure During N95 Respirator Reuse and Extended Use. *JAMA Netw Open.* 2024;7(1):e2353631.

8. Degesys NF, Wang RC, Kwan E, et al. Correlation Between N95 Extended Use and Reuse and Fit Failure in an Emergency Department. *Jama.* 2020;324(1):94-96.

9. Implementing Filtering Facepiece Respirator (FFR) Reuse, Including Reuse after Decontamination, When There Are Known Shortages of N95 Respirators. Updated October 19, 2020. CDC. . <https://web.archive.org/web/20220502152932/https:/www.cdc.gov/coronavirus/2019-ncov/hcp/ppe-strategy/decontamination-reuse-respirators.html#ref1>.

10. 3M™ Disposable Respirator 1870+, N95. Technincal Data Sheet. . <https://multimedia.3m.com/mws/media/1538980O/3m-disposable-respirator-1870-technical-data-sheet.pdf>. Accessed 06/12/2023.

11. 3M™ Disposable Respirator 1860, 1860S, N95. Technical Data Sheet. <https://multimedia.3m.com/mws/media/1538979O/3m-disposable-respirator-1860-1860s-technical-data-sheet.pdf>. Accessed 06/12/2023.

12. 3M™ Aura™ Particulate Respirator, 9205+, N95. Technical Specification Sheet. <https://multimedia.3m.com/mws/media/1837128O/3m-aura-particulate-respirator-9205-n95-technical-specification-sheet.pdf>. Accessed 06/12/2023.

13. 3M™ Particulate Respirator 8210, N95. Technical Specification Sheet. <https://multimedia.3m.com/mws/media/1425070O/3m-particulate-respirator-8210-n95-technical-specifications.pdf>. Accessed 06/12/2023.

14. O&M Halyard Inc #46727/46767. Product Specifications. <https://mms.mckesson.com/product/286249/OM-Halyard-Inc-46727>. Accessed 06/12/2023.

15. O&M Halyard Inc #46827. Product Specifications. <https://mms.mckesson.com/product/286250/OM-Halyard-Inc-46827>. Accessed 06/12/2023.

16. Halyard N95 Particulate Filter Respirator - N95M76727/M76827. <https://www.mhcaremedical.com/product/halyard-n95-particulate-filter-respirator-and-surgical-masks>. Accessed 06/12/2023.

17. Aijaz J, Naseer F, Dojki M, et al. Duration of respiratory sample stability at -80ºC for SARS-CoV-2 PCR. *Pak J Med Sci.* 2022;38(2):393-398.
